# The development of memory maintenance strategies: training cumulative rehearsal and interactive imagery in children aged between 5 and 9

**DOI:** 10.3389/fpsyg.2015.00524

**Published:** 2015-05-01

**Authors:** Sadie Miller, Samantha McCulloch, Christopher Jarrold

**Affiliations:** School of Experimental Psychology, University of BristolBristol, UK

**Keywords:** rehearsal, interactive imagery, training, development, memory maintenance

## Abstract

The current study explored the extent to which children above and below the age of 7 years are able to benefit from either training in cumulative rehearsal or in the use of interactive imagery when carrying out working memory tasks. Twenty-four 5- to 6-year-olds nd 24 8- to 9-year olds were each assigned to one of three training groups who either received cumulative rehearsal, interactive imagery, or passive labeling training. Participants’ ability to maintain material during a filled delay was then assessed, and the nature of the distraction that was imposed during this delay was varied to shed further light on the mechanisms that individuals used to maintain the memoranda in working memory in the face of this distraction. The results suggest that the rehearsal training employed here did improve recall by virtue of encouraging rehearsal strategies, in a way that was not observed among participants receiving interactive imagery training. The fact that these effects were not mediated by age group counts against the view that younger individuals are either unable to rehearse, or show impoverished verbal serial recall because they do not spontaneously engage in rehearsal.

## Introduction

Working memory refers to the ability to store and manipulate information over brief periods of time (Baddeley and Hitch, 1974; Kane and Engle, 2002). Working memory performance is often measured using complex-span tasks, which consist of interleaved storage and processing demands (Daneman and Carpenter, 1980; Case et al., 1982), and scores on such tasks have been found to be predictive of numerous abilities, including academic performance (Gathercole et al., 2003), indicators of intelligence (e.g., Kane and Engle, 2003), and classroom behavior (Gathercole et al., 2006). Given the association between working memory performance and these important childhood aptitudes, understanding the structure and development of the working memory system has important real-world implications.

One clear constraint on working memory performance is short-term memory, as while working memory tasks tap the ability to maintain information *in the face of distraction*, one’s ability to hold material in correct serial order does play an important role in determining task performance (Engle et al., 1999; Bayliss et al., 2003; Alloway et al., 2006). Indeed, individual differences studies have shown that children’s working memory performance draws on either their verbal or visuo-spatial short-term memory capacities, depending on whether the task involves the maintenance of either verbal or visual information (Bayliss et al., 2003; Alloway et al., 2006). In line with this, developmental changes in short-term memory capacity have been shown to underpin at least some of the age-related changes in working memory performance (Bayliss et al., 2005).

This raises the question of what causes developmental increases in short-term memory capacity, and one suggestion in the literature is that age-related changes in the use of rehearsal mediate improvements in short-term memory (e.g., Dempster, 1981; Baddeley, 1986; Hitch et al., 1989a; Gathercole, 1998). For example, Hitch et al. (1989a, p. 347) suggested that “the development of rehearsal strategies contributes to the increase in (verbal short-term memory) performance with age.” According to Baddeley’s (1986) highly influential model of working memory, articulatory rehearsal is responsible for both the phonological coding of non-verbal material and the strengthening of phonological memory traces to prevent them from suffering from rapid decay. As a result, potential evidence for the use of subvocal rehearsal comes from the fact that adults recode visually presented information in short-term memory tasks into a phonological form, as evidenced by a subsequent phonological similarity effect with relatively poorer recall of phonologically confusable material (Conrad and Hull, 1964). In a similar vein, poorer recall of words of a longer as opposed to shorter spoken duration – the word length effect (Baddeley et al., 1975) – is also seen by many as a marker of rehearsal (though see Lewandowsky and Oberauer, 2008). Baddeley’s (2000) model also includes within it the visuo-spatial sketchpad, the system that supports the temporary storage of visuo-spatial information. It has been suggested that visual information can also be ‘rehearsed’ in some non-vocal form (Read, 1979). In an extension of this model, Logie (1995, 2011) suggested that the visuospatial sketchpad might be best viewed as consisting of two sub-systems, a visual cache that stores images and an inner scribe that engages in a form of spatially mediated rehearsal of this material. Alternatively, a process of interactive imagery, that involves creating images that depend on semantic links to long-term memory, might also support maintenance of visual material that might otherwise be lost from visuo-spatial short-term memory (cf. Bower, 1970).

There is evidence that children become increasingly more strategic with age and so might be expected to make more use of memory maintenance strategies such as rehearsal as they get older (e.g., Ornstein et al., 1975; Dempster, 1981; Palmer, 2000; Lehmann and Hasselhorn, 2007; though see Jarrold and Hall, 2013). A key question for those who believe in age-related change in these memory maintenance activities is whether such a change is qualitative or quantitative in nature. Many authors have reported an absence in young children of both phonological similarity effects for visually presented material and of word length effects (e.g., Henry, 1991; Hitch et al., 1991; Henry et al., 2000, 2012), leading to the dominant view that children undergo a qualitative shift in their use of sub-vocal rehearsal at around 7 years of age (e.g., Flavell et al., 1966) or slightly younger (Henry et al., 2012). The converse effect has been observed in developmental studies of visuo-spatial short-term memory, in that children younger than 7 tend to show a clear and detrimental effect of any visual similarity that exists between visually presented items (e.g., Brown, 1977; Palmer, 2000; though see Henry et al., 2012), that is not apparent in older individuals (Hitch et al., 1988, 1989b; though see Palmer, 2000). This has led to the suggestion that young children maintain visually presented information in a visual format, while older individuals tend to recode such information into a phonological form (see Palmer, 2000); again, such a claim is potentially consistent with the view that a qualitative change in the use of rehearsal, which might support recoding, occurs around 7 years.

However, more recent work has questioned the assumption that the use of sub-vocal rehearsal undergoes a qualitative change with age and is absent in young children. There is evidence that reliable effects of phonological similarity for visually presented material can be observed in young children provided that studies are sufficiently powered to detect these (Al-Namlah et al., 2006; Jarrold and Citroën, 2013). It might be argued that this reflects the fact that not all children acquire rehearsal behavior at exactly the same age, with the result that a qualitative change in each individual is spread over an age range within a sample. While this may be true, the profile of developmental change in short-term memory performance is arguably more linear across ages than even this account would presumably predict (Gathercole, 1999). An alternative is that developmental changes in the use of recoding, and by implication sub-vocal rehearsal, may be quantitative rather than qualitative (Tam et al., 2010). It is also possible that young children’s apparent lack of use of sub-vocal rehearsal is constrained by their relatively low verbal short-term memory capacity, and that children of any age can engage in verbal rehearsal provided that the list of to-be-remembered items does not exceed that capacity (Jarrold and Hall, 2013).

There is therefore a lack of consensus within the literature as to the nature of the development of sub-vocal rehearsal in children, and a consequent need to clarify the role of rehearsal in working memory development. However, the on-line use of sub-vocal rehearsal is very difficult to measure (Tehan and Lalor, 2000), and it has therefore been suggested that training the use of rehearsal is a particularly informative way of determining whether individuals are able to benefit from it or not (Huttenlocher and Burke, 1976). This study therefore attempted to train younger (5–6-year-old) and older (7–8-year-old) children to use cumulative sub-vocal rehearsal, that is, the covert repetition of verbal material in correct serial-order. In addition, a separate group of children in these two age groups were given training in interactive imagery in which they were instructed to visually imagine all the previously presented items being joined together and interacting with one another (Bower, 1970). This allowed us to determine whether younger, as opposed to older, children were more receptive to training with a visually based rather than a verbal maintenance strategy. A final sub-sample of children of each age group received a third form of training that was designed to promote neither cumulative rehearsal nor interactive imagery strategies, and which therefore served as an ‘active control’ condition. This involved teaching participants to name only the just-presented item to themselves after it was presented. Labeling of this form is thought to be a passive and primitive form of maintenance that, in developmental terms, occurs prior to the onset of cumulative rehearsal (Lehmann and Hasselhorn, 2007). In sum, children in each age group were assigned to one of three separate training conditions: rehearsal training, imagery training, and control training.

Previous rehearsal training studies have produced mixed results (see Gathercole and Alloway, 2008), but these studies have tended to focus solely on immediate memory span as the outcome measure. Importantly, in this study we instead investigated the processes that children were using to maintain information following training by exploring the extent to which distracting tasks imposed between presentation and recall of to-be-remembered information impaired recall (cf. Rai and Harris, 2013). Specifically, the key outcome measures were performance on two Brown–Peterson tasks (Brown, 1958; Peterson and Peterson, 1959) in which to-be-remembered material had to be maintained for subsequent ordered recall across a filled delay. In all tasks memoranda were simultaneously presented in both auditory and visual formats. This was done partly to ensure correct encoding of item identity, which cannot be assumed with just pictorial presentation, but also to allow participants to elect to maintain this information in either a phonological or a visuo-spatial form. The two Brown–Peterson tasks differed only in the type of distraction that was imposed during the delay interval, with one imposing verbal distraction by asking participants to carry out rhyme judgments, and the other imposing visual distraction by requiring visual decisions. Importantly, *exactly* the same stimuli were used for the processing phase of both the ‘verbal distraction Brown–Peterson’ and the ‘visual distraction Brown–Peterson’ task in order to maximize their comparability (cf. Jarrold et al., 2011).

With this design we were able to determine whether type of short-term memory training interacted with the extent to which participants’ working memory was affected by either verbal or non-verbal (visual) distraction. Sub-vocal rehearsal is assumed to use speech-planning mechanisms, albeit at a covert level, thus any task that also requires these mechanisms should prevent rehearsal (cf. Hudjetz and Oberauer, 2007). Consequently, verbal distraction in a Brown–Peterson task should lead to poorer recall if participants are attempting to maintain the memoranda using a sub-vocal rehearsal strategy. In contrast, if integrative imagery training leads to an image being stored and rehearsed in a visual cache (Logie, 1995), then more forgetting should be caused by visual distraction in a Brown–Peterson task when individuals are using this approach to memory maintenance. We therefore predicted that if participants of any age were able to acquire the use of sub-vocal rehearsal via rehearsal training, then they would show superior recall than their counterparts in the control training group on the visual distraction Brown–Peterson task, while perhaps also doing less well than these control participants on the verbal distraction Brown–Peterson task. Conversely, any participants in the imagery training group who acquired an interactive imagery strategy would be expected to outperform children of the same age in the control training group on the verbal distraction Brown–Peterson task, whilst potentially performing less well than these individuals on the visual distraction Brown–Peterson task. Finally, examining whether age interacted with these effects would allow us to determine whether younger children benefit more, less, or to the same extent as older children from these different training regimes.

## Materials and Methods

### Participants

A total of 24 participants from School Year 1 (10 female, 14 male, *M*_age_ = 73 months, SD = 3.49), and 24 from School Year 3 (10 female, 14 male, *M*_age_ = 97 months, SD = 3.53), of a local primary school took part in the study. Eight children from each year group were allocated to each of the three training conditions (i.e., *n* = 16 for total number of children in each training condition). Full informed parental consent was obtained for each participant.

### Procedure

The experiment consisted of three phases. Participants were taken to a quiet room to complete each experimental phase on three occasions each no more than 5 days apart. The experimenter recorded participants’ memory recall manually. There were no time constraints on memory recall in any task. In phase 1 participants completed a simple span task. Participants were then allocated to a training condition based on this simple span score to ensure that the different training condition groups were equated for initial verbal short-term memory performance. Phases 2 and 3 were identical in structure, and included 3 ‘games’; a practice of the processing-task, training, and a Brown–Peterson task. The type of distracting processing (verbal or visual) embedded in the Brown–Peterson task was counterbalanced across phases 2 and 3.

Memory stimuli consisted of images deemed appropriate/familiar to young children. Images were 160 × 160 pixels in size, and were presented in black squares of size 7 cm × 7 cm that were arranged diagonally on a laptop. The number of squares shown represented the amount of items to be presented on any trial. Sound files (a male voice) associated with the images were created to be played simultaneously with the visual presentation of an ‘item.’ All items were randomized across experimental phases with less than one repetition across conditions. The experiment was created using ‘Live Code’ software.

#### Phase 1 Simple Span Task

Stimuli were 60 items, which appeared for 1 s with a 1 s inter-stimulus interval. Subsequent to the final item, participants saw empty squares corresponding to the number of items in the just-presented list prompting immediate serial-recall. Participants were instructed to recall the list verbally. There were 15 trials ordered sequentially by list-length (2/3/4/5/6) with three trials at each list length. All participants started at list-length 2, and the task ceased following failure to recall all items in a single trial at a given list-length at least once in correct serial-order.

#### Phase 2 and 3 Processing Task Practice

**FIGURE 1:**
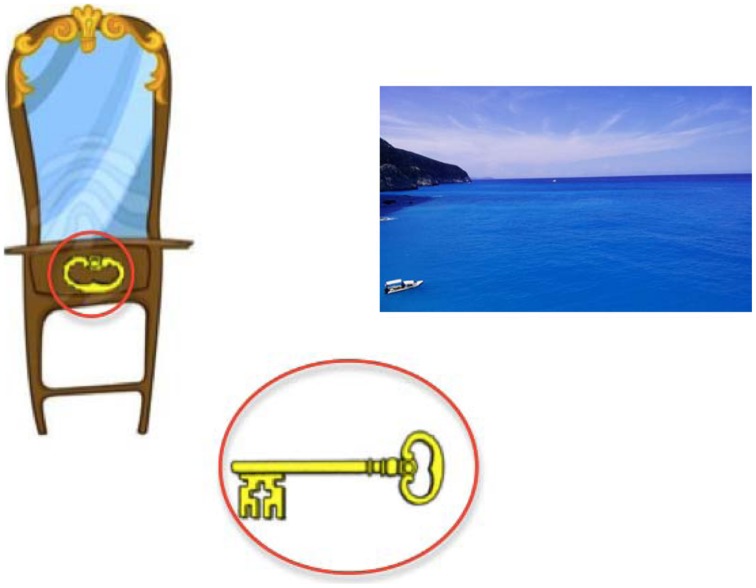
**Example processing stimuli used in phases 2 and 3 (practice processing and Brown–Peterson tasks)**. Participants judge which of the two upper images either rhymes with (verbal processing) or shares a visual feature with (visual processing) the lower central target. Here, for ease of identification, the shared visual features are highlighted by the red circles, which would not be present in the actual stimulus.

Participants were introduced to the ‘puzzle task’ at the start of phases 2 and 3, which served as a practice for subsequent tasks. Each processing task ‘puzzle’ was compiled of three images (see **Figure 1**) with the same images being used for both verbal and non-verbal versions of the task. The lower, central image formed the ‘target,’ which either rhymed (verbal task) or shared a visual feature (non-verbal task) with one of the two upper images. Participant responses and reaction times (RTs) were recorded by the experimental software after the participant pressed either the ‘z’ or ‘/’ key to select the left and right image, respectively. Participants viewed a practice demonstration then, completed 10 trials.

#### Phase 2 and 3 Strategy Training

Training consisted of three between-participant conditions (control/rehearsal/imagery), and was presented in both phases 2 and 3. Stimuli were two sets of 18 items, but participants were only ‘trained’ at list-lengths determined by their performance in the simple span task from phase 1 (with the first trained list length being the longest list-length successfully recalled in the phase 1 simple span task). Participants engaged in three training trials; in each items were presented consecutively for 1 s, with a 2 s interval between each item. The first trial demonstrated the training technique. Trial 2 allowed participants to practice the trained technique at a list-length equivalent to their maximum ‘span’ from the simple span task of phase 1, while trial 3 presented a list at list-length span +1.

In the control training condition to-be-remembered items were presented individually with a female voice played in the inter-stimulus interval that repeated only the name of the just-presented item, encouraging labeling. In contrast, in the rehearsal training condition sound files were presented in a cumulative fashion, where each black square, representing the number of to-be-remembered memoranda being ‘rehearsed’ at the point, turned red to demonstrate the cumulative nature of this form of rehearsal. Intervals between stimuli were therefore filled with a female voice that labeled all previously presented items in their correct order, encouraging rapid cumulative rehearsal. The imagery training condition re-presented images of all the previously presented items in between the presentation of each stimulus item. To encourage integrative imagery no sound files were presented during these inter-stimulus intervals, but rather a compilation of all previous images appeared in the bottom left corner of the screen in between the presentation of each stimulus.

Participants were not explicitly instructed to verbally rehearse or to use interactive imagery at the start of the rehearsal or imagery training conditions. However, prior to trial 1 they were told that the “computer would show them a great way of remembering things.” After trial 1 they were asked to tell the Experimenter “how the computer had told them to remember,” and before both trials 2 and 3 they were encouraged to “use that way to remember” the material that was about to be presented.

#### Phase 2 and 3 Brown–Peterson Task

**FIGURE 2:**
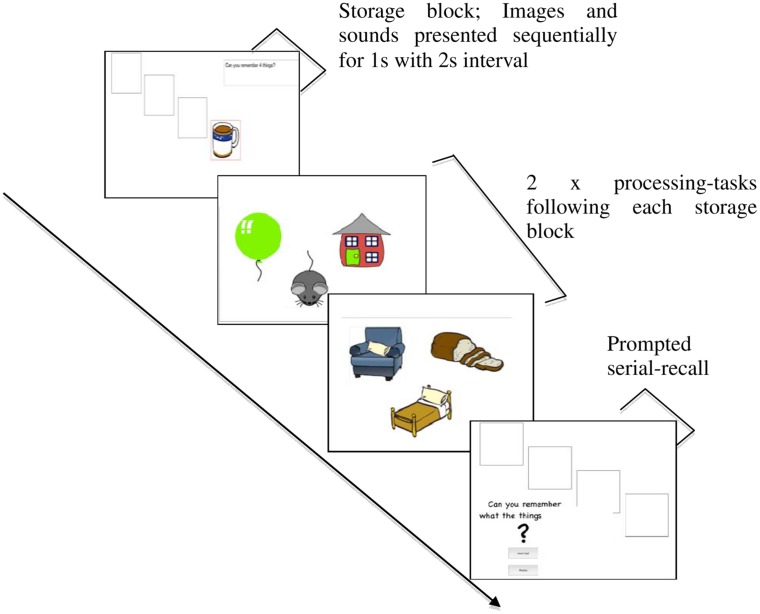
**An example trial from a Brown–Peterson task at list-length 4**.

Two Brown–Peterson like tasks (verbal distraction, visual distraction) were created, one for each phase of the experiment, where blocks of to-be-remembered items were initially presented followed by a subsequent block of distracting processing (the puzzle-task), see **Figure 2**. Memory stimuli were drawn from two sets of 60 items. This allowed for 15 trials in each task, three at each of list-lengths 2–6. Trials of different list-lengths were presented in a pseudo-random order, rather than being blocked, and all participants completed all trials regardless of their simple span score from phase 1. Memory items were presented individually for 1 s, with a 2 s interval between them, to allow time for rehearsal. Immediately after presentation of the last memory item, two puzzle task processing items were presented. The participant was told in advance whether to perform verbal or visual processing of these puzzle tasks in that particular phase (type of distraction was blocked within, and counterbalanced across, phase). Processing responses and processing RTs were recorded. Following completion of the second processing task a new screen appeared prompting spoken recall of the previously presented memoranda.

Participants were not explicitly asked to report their use of maintenance strategies during these tasks, because of concerns about the reliability of self-report of strategy use among children of this age. However, spontaneous verbalisation was observed among some participants in both the control and rehearsal training groups. Our anecdotal impression was that verbal utterances were less common among individuals in the imagery training group.

## Results

One participant in the control training condition (in Year 1) was removed due to failing to complete the second experimental phase. For the remaining participants, partial credit scores (PCS, Conway et al., 2005) were calculated for the simple-span and Brown–Peterson tasks by summing the number of correct items recalled, proportioned against the trial list-length, across all trials.

### Training Condition Allocation

First, to confirm that participants within the three training conditions were equated for baseline levels of verbal short-term memory ability, a 2 (year-group: 1/3) × 3 (training: control/rehearsal/imagery) independent analysis of variance (ANOVA) was performed on the PCS obtained from the simple-span task. There was an unsurprisingly significant main effect of year-group *F*(1,41) = 10.61, *p* = 0.002, MSE = 4.43, $\eta_{p}^{2}$ = 0.206, where younger participants recalled less than older participants (Year 1: *M* = 8.13, SD = 1.95; Year 3: *M* = 10.11, SD = 2.10). Importantly there was no significant effect of training condition, indicating groups were successfully equated for simple-span performance, *F*(2,41) = 0.20, *p* = 0.823, MSE = 4.43, $\eta_{p}^{2}$ = 0.009. The age x training condition interaction was also clearly non-significant *F*(2,41) = 0.15, *p* = 0.861, MSE = 4.43, $\eta_{p}^{2}$ = 0.007.

### Processing Task Performance

Another preliminary analysis examined the relative difficulty of the distracting processing tasks employed in the practice processing task and the Brown–Peterson tasks in Phases 2 and 3. **Table 1** shows summary statistics of speed and accuracy data from the practice processing tasks, which involved no concurrent presentation of to-be-remembered items and so provides a meaningful index of processing difficulty.

**Table 1:** **Descriptive statistics for reaction times (to correct trials only) and accuracy during the practice ‘puzzle tasks’ of phases 2 and 3 that involved no concurrent memory load**.

	Task (type of processing)
	Verbal			Visual		
	***M***	**SD**	**Range**	***M***	**SD**	**Range**

****RT (ms)****
Year 1	7745	3064	4652–11429	6225	2235	2675–11308
Year 3	6325	2719	3316–9048	4217	1934	2167–9379
**Total**	7020	2950		5200	2300	
**Accuracy**
Year 1	0.85	0.14	0.70–1.00	0.92	0.10	0.70–1.00
Year 3	0.92	0.09	0.80–1.00	0.92	0.09	0.80–1.00
**Total**	0.89	0.12		0.91	0.09	

First, a 2 (year: 1/3) × 2 (processing: verbal/visual) mixed ANOVA was conducted on processing RTs (averaged across just correct trials). There was a significant main effect of processing type, *F*(1,45) = 11.17, *p* = 0.002, MSE = 5716471, $\eta_{p}^{2}$ = 0.199, where verbal processing took longer than visual processing. Further, there was a significant main effect of year group, *F*(1,45) = 7.60, *p* = 0.008, MSE = 7869790, $\eta_{p}^{2}$ = 0.144, as Year 3 children were significantly faster than Year 1 individuals, but no significant interaction between factors, *F*(1,45) = 0.67, *p* = 0.419, MSE = 5716471, $\eta_{p}^{2}$ = 0.046.

A further, identical, 2 × 2 mixed ANOVA was conducted on participants’ average accuracy score for the processing decisions in the practice processing task. There were no significant main effects of processing type, *F*(1,44) = 2.68, *p* = 0.109, MSE = 0.010, $\eta_{p}^{2}$ = 0.057, or of year group, *F*(1,44) = 1.73, *p* = 0.189, MSE = 0.013, $\eta_{p}^{2}$ = 0.073, and no significant interaction between these factors, *F*(2,42) = 1.07, *p* = 0.353, MSE = 0.010, $\eta_{p}^{2}$ = 0.015.

In sum, despite comparable accuracy levels, as verbal processing decisions took significantly longer to make than did visual processing decisions, it cannot be assumed the two distraction tasks were equated for difficulty. However, a further analysis of these RT data that included the additional between-participants factor of training condition showed no significant interaction between processing task type and training condition, *F*(2,44) = 0.94, *p* = 0.400, MSE = 5690872.76, $\eta_{p}^{2}$ = 0.041. In other words, regardless of training condition allocation, all participants took significantly longer to complete the verbal distraction task relative to the visual distraction task.

### Brown–Peterson Task Performance

A preliminary analysis showed no obvious practice effect on recall performance in the Brown–Peterson tasks, in that recall on the first task (regardless of whether it involved verbal or visual processing) was not significantly different from that on the second task (regardless of whether it involved visual or verbal processing), *F*(1,46) = 0.88, *p* = 0.354, MSE = 5.59, $\eta_{p}^{2}$ = 0.019.

**Table 2** summarizes the performance of the sample on the two versions of the Brown–Peterson task. To investigate the effect of training condition on subsequent performance on these Brown–Peterson tasks, a 3 (training: control/rehearsal/imagery) × 2 (year-group: 1/3) × 2 (processing task: verbal/visual distraction) mixed ANOVA was conducted on PCS obtained from these tasks. There was a significant main effect of year-group, *F*(1,41) = 35.25, *p* < 0.001, MSE = 7.84, $\eta_{p}^{2}$ = 0.462, where older participants recalled more overall. The main effect of training condition was also significant, *F*(2,41) = 8.60, *p* = 0.001, MSE = 7.84, $\eta_{p}^{2}$ = 0.296. This reflected lower average PCS in the control condition than either the rehearsal, *F*(1,27) = 8.89, *p* = 0.006, MSE = 9.35, $\eta_{p}^{2}$ = 0.248, or the imagery, *F*(1,27) = 21.64, *p* < 0.001, MSE = 118.16, $\eta_{p}^{2}$ = 0.445, training conditions. Similarly the effect of processing task was significant *F*(1,41) = 52.13, *p* < 0.001, MSE = 2.49, $\eta_{p}^{2}$ = 0.560, due to poorer recall on the verbal distraction Brown–Peterson task. However, this effect was qualified by a significant interaction between the factors of processing task and training condition, *F*(2,41) = 5.41, *p* = 0.008, MSE = 2.49, $\eta_{p}^{2}$ = 0.209.

**Table 2:** **Descriptive statistics for recall performance (partial credit score, PCS) in the Brown–Peterson tasks involving either verbal or visual distraction by year group and for each training condition**.

	Task (type of processing)
	Verbal			Visual		
	***M***	**SD**	**Range**	***M***	**SD**	**Range**
**Control**
Year 1	3.41	1.43	1.90–5.70	5.83	1.47	3.50–8.00
Year 3	7.32	2.46	5.12–11.62	9.47	1.96	6.62–12.17
**Total**	5.50	2.82		7.77	2.53	
**Rehearsal**
Year 1	5.66	3.00	1.75–10.48	8.94	2.45	6.00–12.60
Year 3	8.30	3.06	2.87–12.03	12.40	1.90	9.50–14.33
**Total**	6.98	3.23		10.67	2.77	
**Interactive imagery**
Year 1	6.81	3.07	2.25–10.27	8.27	1.62	5.87–11.22
Year 3	10.64	2.15	7.36–13.55	11.38	1.55	9.35–13.60
**Total**	8.73	3.24		9.82	2.22	

All other interactions were non-significant, *F*(2,41) ≤ 0.506, but given the relatively small sample size of this study, coupled with our interest in the mediating effects of age on strategy use, any null interactions involving age were subjected to a further Bayesian analysis that compared the weight of evidence for the null interaction against that for the alternative hypothesis of a significant interaction. These analyses were conducted using the formulas provided by Masson (2011). For the year-group by training condition the posterior probability for the null hypothesis was 0.98, for the year-group by processing task interaction it was 0.87, and for the three-way interaction it was 0.79. According to the criteria provided by Raftery (1995) all three analyses provide ‘positive’ or better support for the null hypothesis, and for the absence of an interaction with age in each case (the evidence for the null hypothesis is ‘strong’ in the case of the year-group by training condition interaction).

**FIGURE 3:**
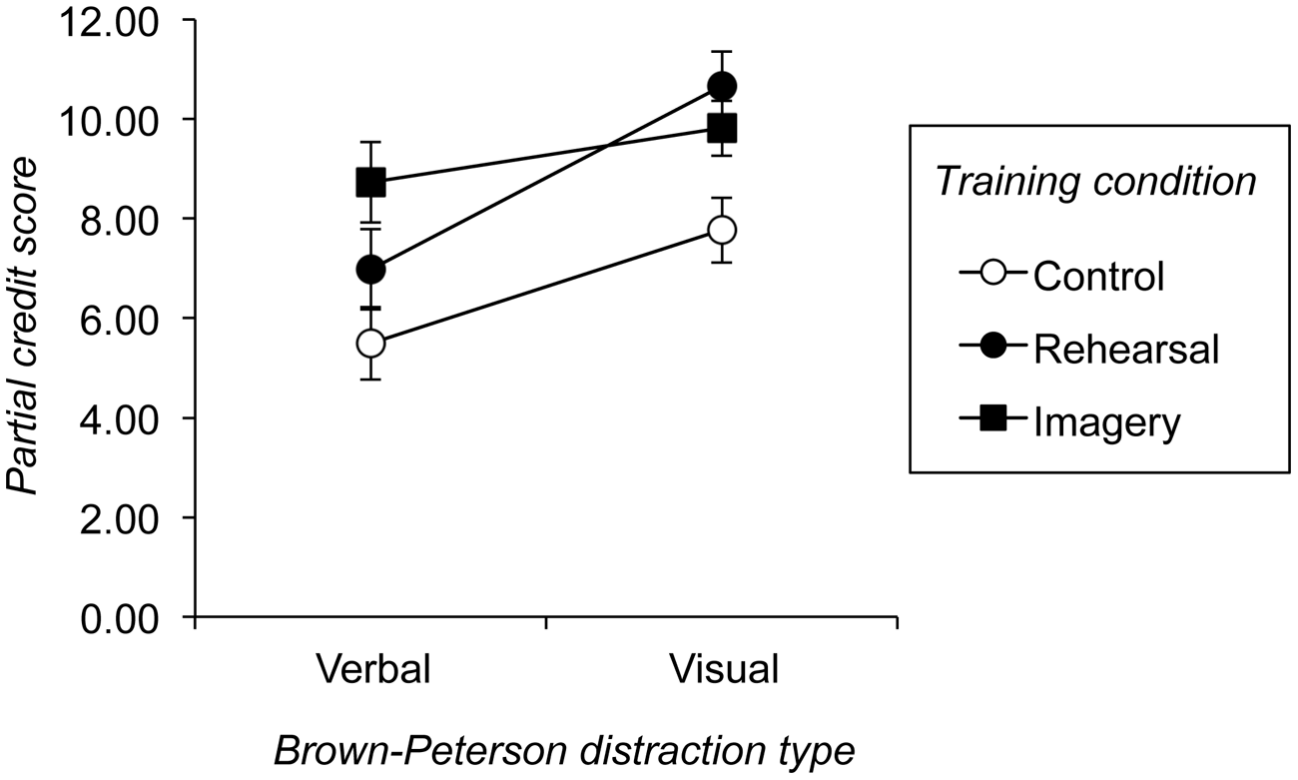
**The interaction between training condition and type of distraction employed in the two Brown–Peterson tasks**. Error bars are ±1 SE.

The interaction of processing task by training condition, shown diagrammatically in **Figure 3**, was explored further by two complementary *post hoc* analyses of simple main effects. First, the effect of training condition was investigated within the verbal and visual distraction Brown–Peterson tasks independently. This particular approach has the advantage of allowing a direct comparison, for each task, between performance of individuals in the rehearsal and imagery training conditions on the one hand, and those in the control training condition on the other. Given that these groups were very well matched for initial levels of short-term memory performance, this analysis provides a means of determining whether rehearsal or imagery training led to a real benefit to performance on either task. Two independent ANOVA’s were conducted on these PCS, each with the single factor of training condition (control/rehearsal/imagery). The effect of training condition was significant when just recall from the verbal distraction Brown–Peterson task was considered, *F*(2,44) = 4.20, *p* = 0.021, MSE = 9.66, $\eta_{p}^{2}$ = 0.160. *Post hoc* analysis showed that there was no significant difference between control and rehearsal training, *F*(1,29) = 1.85, *p* = 0.184, MSE = 9.23, $\eta_{p}^{2}$ = 0.060, or between rehearsal and imagery training *F*(1,30) = 2.32, *p* = 0.137, MSE = 10.45, $\eta_{p}^{2}$ = 0.072. However, there was a significant difference between control and imagery training, *F*(1,29) = 8.73, *p* = 0.006, MSE = 9.26, $\eta_{p}^{2}$ = 0.231, as imagery training led to significantly greater recall. The effect of training condition was also significant when recall from the visual distraction Brown–Peterson task was examined, *F*(2,44) = 5.40 , *p* = 0.008, MSE = 6.33, $\eta_{p}^{2}$ = 0.197. However, in this case, there was a significant difference between control and rehearsal training *F*(1,29) = 9.22, *p* = 0.005, MSE = 7.06, $\eta_{p}^{2}$ = 0.241, where rehearsal training led to greater recall. Imagery training also led to significantly greater recall than control training, *F*(1,29) = 5.77, *p* = 0.023, MSE = 5.60, $\eta_{p}^{2}$ = 0.166, but not relative to rehearsal training *F*(1,30) = 0.92, *p* = 0.345, MSE = 6.30, $\eta_{p}^{2}$ = 0.030.

Second, the effect of processing task was explored within each training condition separately in order to determine the extent to which individuals in a given training group were differentially affected by verbal or visual distraction. Although this obviously duplicates aspects of the previous analysis of simple main effects, in the sense that it focuses on the same set of data shown in **Figure 3**, it provides a means of gauging the type of maintenance method used by a given group that is not dependent on comparisons with other individuals (i.e., a within-participant comparison). Three separate mixed ANOVAs were therefore conducted on the PCS of participants in each training condition, each with the single factor of processing type (verbal/visual distraction). Participants in the control training condition, showed a significant effect of processing task, *F*(1,14) = 34.18, *p* < 0.001, MSE = 1.14, $\eta_{p}^{2}$ = 0.709, as did participants in the rehearsal training condition, *F*(1,15) = 70.17, *p* < 0.001, MSE = 1.55, $\eta_{p}^{2}$ = 0.824. In both cases Brown–Peterson recall was poorer when the task involved verbal distraction. The main effect of processing task on the Brown–Peterson PCS of participants in the visual training condition was non-significant, *F*(1,15) = 2.19, *p* = 0.160, MSE = 4.38, $\eta_{p}^{2}$ = 0.127.

The immediately preceding post-hoc analysis shows that participants in both the control and rehearsal training conditions were more affected by verbal than visual distraction. However, the size of this ‘distraction effect’ was larger in the latter case. Indeed a further analysis that compared the size of the distraction effect across just these two training conditions produced a significant interaction, *F*(1,27) = 4.56, *p* = 0.042, MSE = 1.38, $\eta_{p}^{2}$ = 0.145, confirming the reliably greater detrimental effect of verbal distraction on individuals in the rehearsal training condition (see **Figure 3**).

One concern that potentially might affect the interpretation of this analysis is that verbal processing has already been seen to be more difficult than visual processing. To address this worry a subset of participants was selected who were faster at verbal than visual processing. Participants were arranged in order of the magnitude of the difference between their RTs for verbal and visual processing in the processing practice task and those with slower RT’s for verbal processing were successively removed until the overall group mean RT for verbal processing was numerically smaller than that for visual processing (cf. Jarrold et al., 2011). A total of 16 participants were removed in this way to produce a subsample that was numerically faster at verbal than visual processing. An analysis identical to that just described above was conducted on Brown–Peterson task performance, namely a 2 (training: control/rehearsal) × 2 (year: 1/3) × 2 (processing: verbal/visual distraction) mixed ANOVA. There was still significantly poorer Brown–Peterson recall following verbal compared to visual distraction in this selected subsample, *F*(1,25) = 50.477, *p* < 0.001, MSE = 2.014, $\eta_{p}^{2}$= 0.669. Thus a greater detrimental effect of verbal distraction is apparent when the processing task is embedded in the Brown–Peterson paradigm, even when individuals found the verbal and visual processing tasks equally demanding in the absence of any memory load (cf. Jarrold et al., 2011).

## Discussion

The primary aim of this experiment was to clarify whether training memory maintenance strategies leads to improved working memory performance in children of different ages, and, consequently, to investigate the role of subvocal rehearsal and integrative imagery in short-term memory development. This was investigated by training children to use either cumulative rehearsal or integrative imagery to maintain material. Although previous studies have also examined rehearsal training, the current study incorporated a number of design features that, together, made for a novel experiment that has the potential to shed considerable light on the question of when children are able to employ either verbal or visual memory strategies. In particular, in the current study to-be-remembered material was presented in both auditory and pictorial form, and should therefore have been amenable to either verbal or visual maintenance dependent on the child’s ability to implement such strategies. The inclusion of an active control group who received a similar form of training, albeit one that did not train any cumulative approach to memory maintenance, is a further strength, and allows one to determine whether any benefits seen in the other training conditions exceed what would be expected simply as a result of general levels of experimenter engagement and support (see Shipstead et al., 2012). The fact that the three subgroups of children in each training condition were carefully matched for initial level of verbal short-term memory performance also makes the results of any training difficulties easier to interpret. Finally, the use of a Brown–Peterson procedure to assess the effects of memory training is relatively novel, but also highly informative. In the two versions of this task participants were presented with exactly the same stimuli in the ‘distracting’ processing interval. However, the tasks differed in requiring either verbal or visual processing of this potentially distracting information. As noted initially, one would expect individuals using a verbal maintenance strategy to be more detrimentally affected by verbal than visual distraction, with, potentially, the converse pattern seen in participants engaging in some form of visual maintenance strategy (Oberauer et al., 2012).

The results that emerged from the Brown–Peterson tasks showed that the training implemented here, though very brief and transitory, was successful, since individuals in both the rehearsal and imagery training groups had significantly greater recall relative to the participants in the active control group, despite these groups being extremely closely matched for initial levels of recall on the simple-span task. On average participants had significantly poorer Brown–Peterson recall following verbal as opposed to visual distraction. However, despite the use of identical processing materials in these two tasks, the separate processing practice tasks showed that the verbal processing decisions required here were not of equal difficulty to the non-verbal decisions that were made on the same materials; verbal decisions were as accurate, but took significantly longer to make. This raises the possibility that the greater effect of verbal distraction is simply a reflection of the general ‘cognitive load’ associated with this particular task (cf. Barrouillet et al., 2011). Importantly, while this may explain part of this effect, the subsequent analysis of the subsample of individuals who took numerically less time to complete verbal as opposed to visual decisions in the processing practice task also showed a greater effect of verbal distraction in the Brown–Peterson data. Consequently, it does appear that the verbal nature of this distraction, and not just the general cognitive load that it exerts, underpins its specific impact on the memoranda.

Having said this, one key finding that emerged from the analysis of the Brown–Peterson tasks was the reliable interaction between type of distraction and training condition. Individuals in the control training condition were more detrimentally affected by verbal than visual distraction, suggesting either that labeling did encourage use of verbal maintenance strategies or that participants spontaneously prefer such approaches to more visual ones. However, crucially, this detrimental effect of verbal distraction was significantly greater among individuals in the rehearsal training condition. As a result, it is entirely plausible that the superior recall of individuals in this training condition relative to those in the control condition, seen particularly in the visual distraction Brown–Peterson task, reflects the greater use of rehearsal in the former group. In contrast, but in line with our predictions, children who received interactive imagery training were not particularly affected by verbal processing in the verbal distraction Brown–Peterson task, and showed better performance on this version of the task than the other two groups (significantly so in terms of the contrast with participants in the control condition, see **Figure 3**). This result is consistent with the claim that participants in the imagery training condition employed a non-verbal form of memory maintenance instead of the verbal approaches apparent in the other two groups. This suggests that such a form of visually mediated maintenance can be particularly effective in situations where either rehearsal is prevented or where verbal distraction leads to forgetting of verbal representations in working memory.

One potential problem for this suggestion, however, is that children in the interactive imagery training condition were not particularly affected by the imposition of visual processing in the visual distraction Brown–Peterson task; on this task they performed no worse than individuals in the rehearsal training group, and significantly better than those in the control training group. One might argue that the requirement to produce a spoken response at the point of recall in the Brown–Peterson tasks would have encouraged at least some degree of verbal coding in all groups. If so, then perhaps participants in the imagery training condition stored and maintained to-be-remembered items in a dual code (Paivio, 1969, 1971), rather than purely as a visual image. If items were maintained in this form of dual code only by participants in this training group, then this may well have limited the negative effects of visual distraction while allowing this group to perform particularly well when verbal coding was hampered by verbal processing in the verbal distraction Brown–Peterson task. Alternatively, the visual distraction produced by having to match visual features in the visual processing task (cf. **Figure 1**) may have recruited different representational codes from those used to maintain an interactive image, hence explaining the relative lack of visual interference in this group. Although entirely *post ho* c, this explanation is at least consistent with other data showing that the effects of visual distraction on visuo-spatial storage are less robust than the corresponding effects of verbal distraction on verbal short-term memory (Bayliss et al., 2003; Oberauer et al., 2012). For example, interactive imagery may allow for the formation of a representation that has semantic aspects to it (Bjorklund, 1987) that would then be resistant to visual interference (Andrade et al., 2002). Indeed, research has suggested that irrelevant visual information can interfere with image generation, but does not particularly affect subsequent maintenance of these images (Andrade et al., 2002; Zimmer and Speiser, 2002; Borst et al., 2012).

The second key finding that emerged from the analysis of Brown–Peterson performance following memory training was the absence of any meaningful interactions between any of the experimental factors and age group. It is important to note that age effects on recall were certainly observed in this study, both on the phase 1 test of immediate serial recall and on the subsequent Brown–Peterson tasks. Nevertheless, the effects of the type of distraction embedded in the Brown–Peterson tasks, the effect of training condition, and, crucially, the interaction between distraction type and training condition, were not moderated to any noticeable degree by age.

Of course, one must always be cautious when interpreting null effects, and this concern is particularly relevant in a study such as this which has a relatively small sample size (a total sample of 47 participants with the removal of one participant initially, and split across 2 year groups and three training conditions). To address this concern we reported above a set of Bayesian analysis that provided positive or better evidence to support the view that age did not interact with the other factors in the experimental design. Nevertheless, this evidence was only ‘strong’ in the case of the interaction between year group and training condition, and so interpretations of the null interaction between year group, training condition, and type of processing distraction must still be treated with some degree of caution.

The strong evidence for the fact that cumulative rehearsal training and interactive imagery training were both equally effective for Year 1 and Year 3 participants suggests that similar processes constrain working memory performance in these two age groups. As age differences in Brown–Peterson performance were not differentially affected by either rehearsal or interactive imagery training, it appears that younger participants were able to utilize these strategies as effectively as older individuals. For example, the fact that rehearsal training led to significantly greater recall on the visual distraction Brown–Peterson task in all individuals (in other words not to a significantly different extent across age groups) shows that both Year 1 and Year 3 children performed better on this task when trained to rehearse than did their counterparts in the active control condition. In addition, it seems unlikely that more of the younger children were unable to benefit from this, or the interactive imagery, training because if this were the case, then one would expect to see smaller overall benefits from training in the Year 1 than in Year 3 children, which was not observed. It is also worth noting that age differences in performance were not reduced in either the rehearsal or imagery training groups relative to that seen in the active control subgroup, which would have resulted in an interaction between year-group and training condition that was not observed here. This implies that the poorer recall abilities of younger individuals in this sample seen on the phase 1 immediate recall task cannot solely be due to an absence of rehearsal in this younger subgroup. If that were the case, then training rehearsal, which other aspects of the data suggest was successful, would have particularly benefited these younger individuals.

These findings therefore count to some extent against the claim that children below the age of seven do not engage in spontaneous rehearsal. It should be noted that other studies have argued that a qualitative change in rehearsal status might occur at a somewhat earlier age, and perhaps at around the age of the younger individuals assessed here (Henry et al., 2012). If so, then somewhat different results may have been found if an even younger group had been included in the present study. While this is certainly possible, our reading of the current data is that they challenge the view that there is substantial age-related variation in the extent to which children are able to rehearse. In that sense the findings are in line with recent claims that apparent developmental changes in the use of rehearsal are in fact secondary to age-related increases in recall capacity (Jarrold and Hall, 2013; see also Lehmann and Hasselhorn, 2012). If this is true, then rehearsal training will be of potential benefit to children of any age, particularly if individuals are trained on lists that are close to, or only just exceed, their short-term memory capacity, as was the case in the current study. The results of this study also indicate a potential role for training in interactive imagery. This strategy appears to be available to the younger group of children assessed here who used it to good effect, as did older children, when maintaining information in the face of verbal distraction.

## Conflict of Interest Statement

The Guest Associate Editor, Martin Lehmann, declares that, despite having collaborated with author, Christopher Jarrold, the review process was handled objectively. The authors declare that the research was conducted in the absence of any commercial or financial relationships that could be construed as a potential conflict of interest.

## References

[B1] Alloway T. P. Gathercole S. E. Pickering S. J. (2006). Verbal and visuospatial short-term and working memory in children: are they separable? *Child Dev.* 77 1698–1716 10.1111/j.1467-8624.2006.00968.x 17107455

[B2] Al-Namlah A. S. Fernyhough C. Meins E. (2006). Sociocultural influences on the development of verbal mediation: private speech and phonological recoding in Saudi Arabian and British samples. *Dev. Psychol.* 42 117–131 10.1037/0012-1649.42.1.117 16420122

[B3] Andrade J. Kemps E. Werniers Y. May J. Szmalec A. (2002). Insensitivity of visual short-term memory to irrelevant visual information. *Q. J. Exp. Psychol. A* 55 753–774 10.1080/02724980143000541 12188511

[B4] Baddeley A. (2000). The episodic buffer: a new component of working memory? *Trends Cogn. Sci.* 4 417–423 10.1016/S1364-6613(00)01538-2 11058819

[B5] Baddeley A. D. (1986). *Working Memory*. Oxford: Oxford University Press.

[B6] Baddeley A. D. Hitch G. J. (1974). “Working memory,” in *The Psychology of Learning and Motivation* ed. Bower G. (New York, NY: Academic Press), 47–89.

[B7] Baddeley A. D. Thomson N. Buchanan M. (1975). Word length and the structure of short-term memory. *J. Verbal Learn. Verbal Behav.* 14 575–589 10.1016/S0022-5371(75)80045-4

[B8] Barrouillet P. Portrat S. Camos V. (2011). On the law relating processing to storage in working memory. *Psychol. Rev.* 118 175–192 10.1037/a0022324 21480738

[B9] Bayliss D. M. Jarrold C. Baddeley A. D. Gunn D. M. Leigh E. (2005). Mapping the developmental constraints on working memory span performance. *Dev. Psychol.* 41 579–597 10.1037/0012-1649.41.4.579 16060806

[B10] Bayliss D. M. Jarrold C. Gunn D. M. Baddeley A. D. (2003). The complexities of complex span: explaining individual differences in working memory in children and adults. *J. Exp. Psychol. Gen.* 132 71–92 10.1037/0096-3445.132.1.71 12656298

[B11] Bjorklund D. F. (1987). How age changes in knowledge base contribute to the development of children’s memory: an interpretive review. *Dev. Rev.* 7 93–130 10.1016/0273-2297(87)90007-4

[B12] Borst G. Niven E. Logie R. H. (2012). Visual mental image generation does not overlap with visual short-term memory: a dual-task interference study. *Mem. Cogn.* 40 360–372 10.3758/s13421-011-0151-7 21989739

[B13] Bower G. H. (1970). Imagery as a relational organizer in associative learning. *J. Verbal Learn. Verbal Behav.* 9 529–533 10.1016/S0022-5371(70)80096-2

[B14] Brown J. (1958). Some tests of the decay theories of immediate memory. *Q. J. Exp. Psychol.* 10 12–21 10.1080/17470215808416249

[B15] Brown R. M. (1977). An examination of visual and verbal coding processes in preschool children. *Child Dev.* 48 38–45 10.2307/1128878

[B16] Case R. Kurland D. M. Goldberg J. (1982). Operational efficiency and the growth of short-term memory span. *J. Exp. Child Psychol.* 33 386–404 10.1016/0022-0965(82)90054-6

[B17] Conrad R. Hull A. J. (1964). Information, acoustic confusion, and memory span. *Br. J. Psychol.* 55 429–432 10.1111/j.2044-8295.1964.tb00928.x 14237884

[B18] Conway A. R. A. Kane M. J. Bunting M. F. Hambrick D. Z. Wilhelm O. Engle R. W. (2005). Working memory span tasks: a methodological review and user’s guide. *Psychon. Bull. Rev.* 12 769–786 10.3758/BF03196772 16523997

[B19] Daneman M. Carpenter P. A. (1980). Individual differences in working memory and reading. *J. Verbal Learn. Verbal Behav.* 19 450–466 10.1016/S0022-5371(80)90312-6

[B20] Dempster F. N. (1981). Memory span: sources of individual and developmental differences. *Psychol. Bull.* 89 63–100 10.1037/0033-2909.89.1.63

[B21] Engle R. W. Tuholski S. W. Laughlin J. E. Conway A. R. A. (1999). Working memory, short-term memory, and general fluid intelligence: a latent-variable approach. *J. Exp. Psychol. Gen.* 128 309–311 10.1037/0096-3445.128.3.309 10513398

[B22] Flavell J. H. Beach D. R. Chinsky J. M. (1966). Spontaneous verbal rehearsal in a memory task as a function of age. *Child Dev.* 37 283–299 10.2307/1126804 5941895

[B23] Gathercole S. E. (1998). The development of memory. *J. Child Psychol. Psychiatry* 39 3–27 10.1111/1469-7610.00301 9534084

[B24] Gathercole S. E. (1999). Cognitive approaches to the development of short-term memory. *Trends Cogn. Sci.* 3 410–419 10.1016/S1364-6613(99)01388-1 10529796

[B25] Gathercole S. E. Alloway T. P. (2008). *Working Memory and Learning: A Teacher’s Guide*. London: Sage Publications.

[B26] Gathercole S. E. Brown L. Pickering S. J. (2003). Working memory assessments at school entry as longitudinal predictors of National Curriculum attainment levels. *Educ. Child Psychol.* 20 109–122.

[B27] Gathercole S. E. Lamont E. Alloway T. P. (2006). “Working memory in the classroom,” in *Working Memory and Education* ed. Pickering S. (London: Elsevier Academic Press), 219–240 10.1016/B978-012554465-8/50010-7

[B28] Henry L. A. (1991). The effects of word length and phonemic similarity in young children’s short-term memory. *Q. J. Exp. Psychol. A* 43 35–52 10.1080/14640749108400998

[B29] Henry L. A. Messer D. Luger-Klein S. Crane L. (2012). Phonological, visual, and semantic coding strategies and children’s short-term picture memory span. *Q. J. Exp. Psychol.* 65 2033–2053 10.1080/17470218.2012.672997 22512409

[B30] Henry L. A. Turner J. E. Smith P. T. Leather C. (2000). Modality effects and the development of the word length effect in children. *Memory* 8 1–17 10.1080/096582100387678 10820584

[B31] Hitch G. J. Halliday M. S. Dodd A. Littler J. E. (1989a). Development of rehearsal in short-term memory: differences between pictorial and spoken stimuli. *Br. J. Dev. Psychol.* 7 347–363 10.1111/j.2044-835X.1989.tb00811.x

[B32] Hitch G. J. Woodin M. E. Baker S. (1989b). Visual and phonological components of working memory in children. *Mem. Cogn.* 17 175–185 10.3758/BF03197067 2927315

[B33] Hitch G. J. Halliday M. S. Schaafstal A. M. Heffernan T. M. (1991). Speech, ‘inner speech’, and the development of short-term memory: effects of picture-labelling on recall. *J. Exp. Child Psychol.* 51 220–234 10.1016/0022-0965(91)90033-O 2033361

[B34] Hitch G. J. Halliday M. S. Schaafstal A. M. Schraagen J. M. C. (1988). Visual working-memory in young children. *Mem. Cogn.* 16 120–132 10.3758/BF03213479 3352517

[B35] Hudjetz A. Oberauer K. (2007). The effects of processing time and processing rate on forgetting in working memory: testing four models of the complex span paradigm. *Mem. Cogn.* 35 1675–1684 10.3758/BF03193501 18062545

[B36] Huttenlocher J. Burke D. (1976). Why does memory span increase with age? *Cogn. Psychol.* 8 1–31 10.1016/0010-0285(76)90002-5

[B37] Jarrold C. Citroën R. (2013). Reevaluating key evidence for the development of rehearsal: phonological similarity effects in children are subject to proportional scaling artifacts. *Dev. Psychol.* 49 837–847 10.1037/a0028771 22662766

[B38] Jarrold C. Hall D. (2013). The development of rehearsal in verbal short-term memory. *Child Dev. Perspect.* 7 182–186 10.1111/cdep.12034

[B39] Jarrold C. Tam H. Baddeley A. D. Harvey C. E. (2011). How does processing affect storage in working memory tasks? Evidence for both domain-general and domain-specific effects. *J. Exp. Psychol. Learn. Mem. Cogn.* 37 688–705 10.1037/a0022527 21319919

[B40] Kane M. J. Engle R. W. (2002). The role of prefrontal cortex in working-memory capacity, executive attention, and general fluid intelligence: an individual-differences perspective. *Psychon. Bull. Rev.* 9 637–671 10.3758/BF03196323 12613671

[B41] Kane M. J. Engle R. W. (2003). Working-memory capacity and the control of attention: the contributions of goal neglect, response competition, and task set to Stroop interference. *J. Exp. Psychol. Gen.* 132 47–70 10.1037/0096-3445.132.1.47 12656297

[B42] Lehmann M. Hasselhorn M. (2007). Variable memory strategy use in children’s adaptive intratask learning behavior: developmental changes and working memory influences in free recall. *Child Dev.* 78 1068–1082 10.1111/j.1467-8624.2007.01053.x 17650126

[B43] Lehmann M. Hasselhorn M. (2012). Rehearsal dynamics in elementary school children. *J. Exp. Child Psychol.* 111 552–560 10.1016/j.jecp.2011.10.013 22196371

[B44] Lewandowsky S. Oberauer K. (2008). The word-length effect provides no evidence for decay in short-term memory. *Psychon. Bull. Rev.* 15 875–888 10.3758/PBR.15.5.875 18926980

[B45] Logie R. H. (1995). *Visuo-Spatial Working Memory*. Hove: Lawrence Erlbaum Associates.

[B46] Logie R. H. (2011). The functional organization and capacity limits of working memory. *Curr. Dir. Psychol. Sci.* 20 240–245 10.1177/0963721411415340

[B47] Masson M. E. (2011). A tutorial on a practical Bayesian alternative to null-hypothesis significance testing. *Behav. Res. Methods* 43 679–690 10.3758/s13428-010-0049-5 21302025

[B48] Oberauer K. Lewandowsky S. Farrell S. Jarrold C. Greaves M. (2012). Modeling working memory: an interference model of complex span. *Psychon. Bull. Rev.* 19 779–819 10.3758/s13423-012-0272-4 22715024

[B49] Ornstein P. A. Naus M. J. Liberty C. (1975). Rehearsal and organizational processes in children’s memory. *Child Dev.* 46 818–830 10.2307/1128385

[B50] Paivio A. (1969). Mental Imagery in associative learning and memory. *Psychol. Rev.* 76 241–263 10.1037/h0027272

[B51] Paivio A. (1971). *Imagery and Verbal Processes*. New York: Holt, Rinehart, and Winston.

[B52] Palmer S. (2000). Working memory: a developmental study of phonological recoding. *Memory* 8 179–193 10.1080/096582100387597 10889901

[B53] Peterson L. R. Peterson M. J. (1959). Short-term retention of individual verbal items. *J. Exp. Psychol.* 58 193–198 10.1037/h0049234 14432252

[B54] Raftery A. E. (1995). “Bayesian model selection in social research,” in *Sociological Methodology 1995* ed. Marsden P. V. (Cambridge: Blackwell) 111–196.

[B55] Rai M. K. Harris R. J. (2013). The modified Brown-Peterson task: a tool to directly compare children and adult’s working memory. *J. Genet. Psychol.* 174 153–169 10.1080/00221325.2011.653839 23534194

[B56] Read J. D. (1979). Rehearsal and recognition of human faces. *Am. J. Psychol.* 92 71–85 10.2307/1421480

[B57] Shipstead Z. Redick T. S. Engle R. W. (2012). Is working memory training effective? *Psychol. Bull.* 138 628–654 10.1037/a0027473 22409508

[B58] Tam H. Jarrold C. Baddeley A. D. Sabatos-DeVito M. (2010). The development of memory maintenance: children’s use of phonological rehearsal and attentional refreshment in working memory tasks. *J. Exp. Child Psychol.* 107 306–324 10.1016/j.jecp.2010.05.006 20576275

[B59] Tehan G. Lalor D. M. (2000). Individual differences in memory span: the contribution of rehearsal access to lexical memory, and output speed. *Q. J. Exp. Psychol. A* 53 1012–1038 10.1080/713755933 11131811

[B60] Zimmer H. D. Speiser H. R. (2002). The irrelevant picture effect in visuospatial working-memory: fact or fiction? *Psychol. Beitrage* 44 223–247.

